# Influence of smoking on vascular reactivity to cGMP generators in human internal thoracic arteries

**DOI:** 10.1186/2050-6511-16-S1-A93

**Published:** 2015-09-02

**Authors:** Masashi Tawa, Takeshi Kinoshita, Tohru Asai, Tomoaki Suzuki, Takeshi Imamura, Tomio Okamura

**Affiliations:** 1 Department of Pharmacology, Shiga University of Medical Science, Otsu, Shiga 520-2192, Japan; 2 Division of Cardiovascular Surgery, Department of Surgery, Shiga University of Medical Science, Otsu, Shiga 520-2192, Japan

## Background

There are nitric oxide (NO)-sensitive and -insensitive forms of soluble guanylyl cyclase (sGC). This balance is shifted to the latter under stress conditions associated with increased production of reactive oxygen species (ROS) [1,2]. The present study investigated whether smoking, a well-documented source of ROS, affects NO-sensitive and -insensitive sGC-mediated effects in human arteries.

## Materials and methods

Mechanical responses of internal thoracic arteries obtained from 29 patients undergoing coronary artery bypass grafting were studied. The patients were divided into 2 groups according to their smoking habits: current or ever-smokers (n=17) and former or never-smokers (n=12). Concentration-response curves for nitroglycerin and BAY 60-2770 were constructed in helically-cut strips pre-contracted with phenylephrine, and the values of potency (pD_2_) were calculated. Relaxations induced by the agonists were presented as relative values to the relaxation caused by 10^-4^ M papaverine. Concentration-response curves were analyzed using two-way repeated measures analysis of variance and Bonferroni post hoc test. pD_2_ values were compared with unpaired two-tailed Student's t-test.

## Results

**Figure 1:**
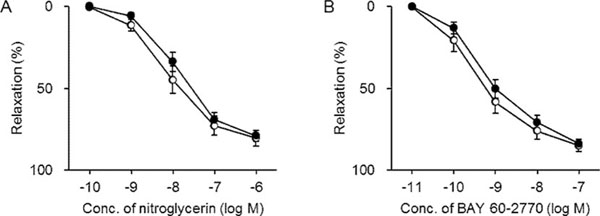
Vasorelaxation induced by nitroglycerin (A) or BAY 60-2770 (B) in internal thoracic arteries from non-smokers (white circle) and smokers (black circle). Each point and bar represents the mean ± SEM of 12 (non-smokers) or 17 (smokers) experiments.

The mean ages of smokers and non-smokers were 70.3 ± 2.1 and 72.3 ± 2.1 years, respectively. The portion of men in smokers (88.2%) was notably higher than that in non-smokers (41.7%). Clinical conditions, laboratory parameters, and medications were similar between the two populations. As shown in Figure 1A, nitroglycerin caused a dose-dependent relaxation, which was not different in the arteries from smokers and non-smokers. pD_2_ values were also identical: 8.03 ± 0.14 (non-smokers) vs. 7.81 ± 0.11 (smokers), p=0.23. Similarly, there was no significant difference in the relaxant potency (pD_2_ values, 9.43 ± 0.22 and 9.15 ± 0.14 for non-smokers and smokers, respectively, p=0.26) and efficacy of BAY 60-2770 (Figure 1B).

## Conclusion

It was demonstrated that smoking is not causal factor for the imbalance between NO-sensitive and -insensitive forms of sGC in human internal thoracic arteries.
